# A web-based appointment system to reduce waiting for outpatients: A retrospective study

**DOI:** 10.1186/1472-6963-11-318

**Published:** 2011-11-22

**Authors:** Wenjun Cao, Yi Wan, Haibo Tu, Fujun Shang, Danhong Liu, Zhijun Tan, Caihong Sun, Qing Ye, Yongyong Xu

**Affiliations:** 1 Department of Health Statistics, School of Preventive Medicine, Fourth Military Medical University, Xi'an 710032, China; 2 Department of Mathematics, Chang Zhi Medical College, Changzhi, Shanxi Province 046000, China; 3 Division of Medical Service, Xijing Hospital, Fourth Military Medical University, Xi'an 710032, China; 4 Tangdu Hospital, Fourth Military Medical University, Xi'an 710038, China

**Keywords:** Web-based appointment system, registration, waiting time, patient satisfaction, non-attendance

## Abstract

**Background:**

Long waiting times for registration to see a doctor is problematic in China, especially in tertiary hospitals. To address this issue, a web-based appointment system was developed for the Xijing hospital. The aim of this study was to investigate the efficacy of the web-based appointment system in the registration service for outpatients.

**Methods:**

Data from the web-based appointment system in Xijing hospital from January to December 2010 were collected using a stratified random sampling method, from which participants were randomly selected for a telephone interview asking for detailed information on using the system. Patients who registered through registration windows were randomly selected as a comparison group, and completed a questionnaire on-site.

**Results:**

A total of 5641 patients using the online booking service were available for data analysis. Of them, 500 were randomly selected, and 369 (73.8%) completed a telephone interview. Of the 500 patients using the usual queuing method who were randomly selected for inclusion in the study, responses were obtained from 463, a response rate of 92.6%. Between the two registration methods, there were significant differences in age, degree of satisfaction, and total waiting time (*P *< 0.001). However, gender, urban residence, and valid waiting time showed no significant differences (*P *> 0.05). Being ignorant of online registration, not trusting the internet, and a lack of ability to use a computer were three main reasons given for not using the web-based appointment system. The overall proportion of non-attendance was 14.4% for those using the web-based appointment system, and the non-attendance rate was significantly different among different hospital departments, day of the week, and time of the day (*P *< 0.001).

**Conclusion:**

Compared to the usual queuing method, the web-based appointment system could significantly increase patient's satisfaction with registration and reduce total waiting time effectively. However, further improvements are needed for broad use of the system.

## Background

Reducing outpatient waiting times has been the focus of a large number of studies [1-3] because waiting and treatment times are usually regarded as indicators of service quality [4]. The Patient's Charter of the UK Government sets a series of standards which state that all patients must be seen within thirty minutes of their appointment time [1]. Outpatient waiting time can be divided into two types: waiting before consultation, and waiting after consultation [5]. Time spent waiting before consultation has attracted much research attention, and can be further separated into waiting time for registration, and waiting time for consultation [6]. Because of China's limited medical resources, long waiting times for registration are common in the health care system, and the registration waiting time is generally much longer than the consultation waiting time. Long registration waiting times for outpatients have already become a long-festering healthcare problem in China [7]. For this reason, our study focuses on registration waiting time only.

In recent years, China has been in the process of implementing health care system reform [8]. The aim of the reform was to provide basic and convenient medical care. Easy access to a doctor is the first step for patients using health services. The traditional registration method (usual queuing method), had unacceptable waiting times, and placed great stress on clinic staff [7]. With the rapid development of the internet over the previous 2-3 years, some hospitals trialed the use of web-based appointment systems (WAS) for outpatients [9]. In 2009, supported by the Ministry of Health, all public tertiary hospitals began to use WAS. However, to date there are few studies about the efficacy of WAS in China. Thus, our study aims to evaluate the efficacy of WAS for outpatients, by comparing waiting times for the WAS and usual queue method, and investigating reasons for not using the WAS.

## Methods

### Study Participants

This work was approved by the ethics committee of Xijing hospital, Fourth Military Medical University. Informed consent was obtained from participants prior to the administration of study measures.

Study participants were recruited from outpatients of Xijing hospital, which is a tertiary hospital in Shaanxi province of China. A two-stage sampling method was adopted. First, from January to December 2010, 10% of all patients registering through the WAS were randomly selected for potential inclusion in the study, giving a total of 5641 patients. For each patient, detailed information including demographic characteristics, appointment, and contact data were obtained from the hospital statistics office. Then, from the selected patients, 500 were randomly selected for a telephone interview. The interview assessed satisfaction with the WAS and the time spent making appointments, and were performed by trained interviewers.

The comparison group completed a questionnaire and comprised 500 randomly selected patients who used the usual queuing method for registration. Interviewers distributed questionnaires to people queuing at registration windows, and supervisors were responsible for the collection of completed questionnaires after registration. The following questions were listed in the questionnaires:

1. What time did you join the queue?

2. What is your reason for not using the web-based registration system?

3. Are you satisfied with the usual queuing method?

4. What time did you register successfully?

The last question was confirmed by the registering nurses.

### Main outcome measures

**Figure 1:**
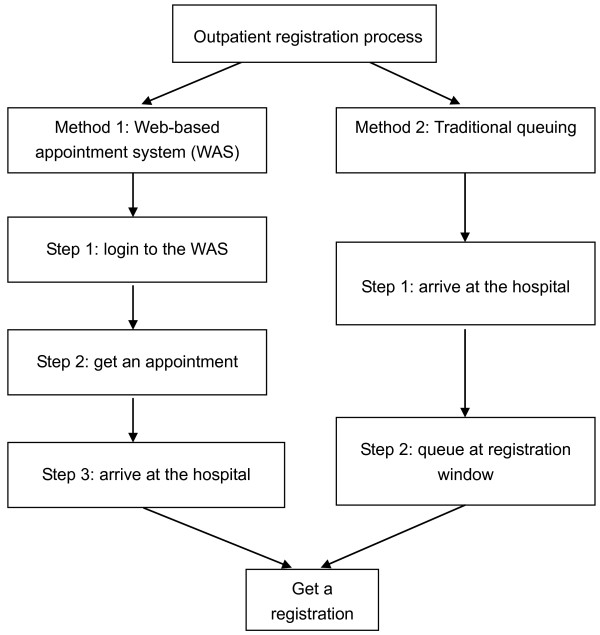
**Flow chart of outpatient registration methods**. "*": WAS refers to web-based appointment system.

We evaluated the performance of two different approaches: the WAS, and the usual queuing method (Figure 1). Using the WAS, patients are given an appointment number. At the designated appointment time, patients arrive at the hospital and get the registration that is allotted to their appointment number. These patients need not queue at the registration window. The patients using the traditional queuing method waste much unnecessary waiting time standing in line at the registration window to ensure a successful registration with a certain physician. The main outcome variables were: invalid, valid, and total waiting time based on the usual queuing method, and appointment-making time based on the WAS. "Invalid waiting time" was defined as the duration of time spent queuing for registration; and "total waiting time" was the total time spent queuing and obtaining registration, and consists of valid and invalid waiting time. Appointment-making time based on the WAS was the duration of time the participant spent making an appointment through the WAS.

### Statistical Analysis

We used SPSS 17.0 for Windows (SPSS Inc, Chicago, Illinois) for all statistical analysis. Continuous variables are presented as means and standard deviations. Categorical variables are presented as frequencies and percentages. The *t*-test, analysis of variance (ANOVA), Mann-Whitney *U *test, and Kruskal-Wallis *H *test were used to compare continuous variables, and the Chi-square test was used to compare categorical variables between the two registration systems. A two-tailed *P*-value < 0.05 was considered statistically significant.

## Results

A total of 5641 people made an appointment using the WAS in the study period, from which 500 were randomly selected for a telephone interview. Responses were obtained from 369 (73.8%) participants. For the usual queuing method group, 463 participants completed a questionnaire while queuing for registration, corresponding to a response rate of 92.6% (463/500).

### The demographic characteristics of participants

Participant gender and residence distributions did not differ between the two registration methods (*P *> 0.05). However, the age distribution was significantly different between the two methods, with the average age of participants being younger in the WAS group (Table 1).

**Table 1:** Demographic factors, degree of satisfaction, and waiting time for participants using usual queuing *vs*. web-based appointment system

Variable	Usual queue method (*n *= 463)	Web-based appointment system (*n *= 369)	*P *value
Age, y, mean ± SD	46 ± 10	34 ± 8	< 0.001
≤ 30 (%)	15 (3.2)	105 (28.5)	< 0.001
30-39 (%)	85 (18.4)	183 (49.6)	_
40-49 (%)	219 (47.3)	54 (14.6)	_
≥ 50 (%)	144 (31.1)	27 (7.3)	_
Gender, n (%)			0.337
Male	162 (35.0)	141 (38.2)	_
Female	301 (65.0)	228 (61.8)	_
Urban residence, n (% in Xi'an zip code)	271 (58.5)	239 (64.8)	0.066
Satisfaction, n (%)	332 (71.7)	181 (49.0)	< 0.001
Average total waiting time, minutes, median (range)	98 (1-811)	7 (3-27)	< 0.001

### Satisfaction and waiting time comparison between the two registration methods

**Figure 2:**
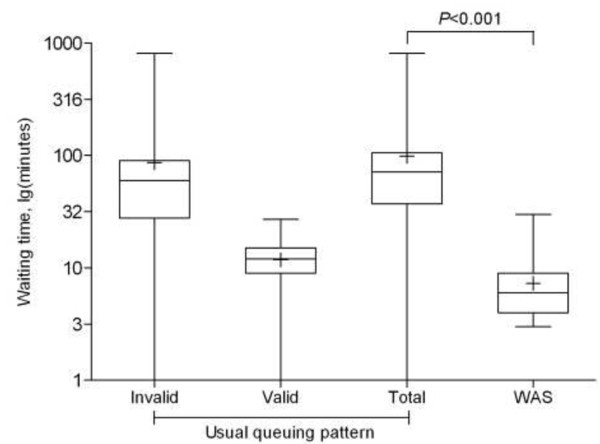
**Box-plot of waiting time for usual queuing *vs*. WAS**. "+": mean values; "WAS": web-based appointment system.

Participants using the WAS reported a higher level of satisfaction with the registration method than those using the usual queuing method (71.7% *vs*. 49.0%, *P *< 0.001). In the usual queuing method, the average invalid waiting time was 86 minutes with a maximum of 13.5 hours; while the average valid waiting time was only 12 minutes (Figure 2). Although the total waiting time of the usual queuing method was significantly longer than that of the WAS method (98 *vs*. 7 minutes, *P *< 0.001) (Table 1), there were no significant differences in valid waiting time between the usual queuing method and the WAS method (12 *vs*. 7 minutes, *P *= 0.321).

### Reasons for not using the WAS

The most common reason given by participants for not using the WAS was that they were unaware of its existence (52.9%) (Table 2). Other factors leading to the non-use of the WAS were that people did not trust the internet (28.1%), and/or lacked the ability to operate a computer (10.4%). Only 8.6% of participants had other reasons, such as that it was casual decision to see a doctor, or that certain physicians had no extra "passports" to be booked.

**Table 2:** Participant reasons for not using the WAS

Reasons	Participants, *n *(%)
Unaware of the online appointment service	245/463 (52.9)
Did not trust the internet	130/463 (28.1)
No computer literacy	48/463 (10.4)
Others	40/463 (8.6)

### Non-attendance following use of the WAS

The overall proportion of non-attendance following making an appointment using the WAS was 14.4%. Non-attendance rates differed among the 30 hospital departments (Kruskal-Wallis *H *test, χ^2 ^= 32.128, *P *< 0.001), and varied between 0.16% and15.7%. Neurosurgery, General surgery, Urology, Obstetrics and Gynecology, and Ophthalmology had the highest rates of non-attendance (Table 3). Non-attendance was higher on Mondays than on any other day (*P *< 0.001). During daytime, the non-attendance rate between 1 pm and 4 pm was higher than the rate between 8 am and 11 am (24.6% *vs*. 10.7%, *P *< 0.001) (Table 3).

**Table 3:** Non-attendance rates in different subgroups of participants using the WAS (*n *= 5 641)

Variable	Non-attendance, no. (%)	*P *value
Overall	812/5641 (14.4)	_
Department		< 0.001
Neurosurgery	144/829(28.6)	_
General surgery	14/69(28.1)	_
Urology	20/112(17.9)	_
Obstetrics and Gynecology	140/787(17.8)	_
Ophthalmology	73/416(17.5)	_
Other	421/3428(12.3)	_
Day of the week		< 0.001
Sunday	6/51(0.9)	_
Monday	224/1230(18.2)	_
Tuesday	160/1241(12.9)	_
Wednesday	146/919(15.9)	_
Thursday	126/965(13.1)	_
Friday	125/970(12.9)	_
Saturday	25/265(9.6)	_
Hours of the day		< 0.001
08:00 to 11:00	442/4138(10.7)	_
13:00 to 16:00	370/1503(24.6)	_

## Discussion

One of the greatest complaints of the Chinese public is the amount of time it takes to queue for outpatient registration in China. From our investigation we noted that some participants even waited in line all night (13.5 hours) to ensure a registration with a certain physician. To effectively reduce registration waiting times in the usual queuing method is an urgent issue that needs to be resolved. This study demonstrates that that using a WAS could substantially increase patients' satisfaction with outpatient registration. Although some time is still needed to make appointments using the WAS, it can significantly reduce total waiting time, especially invalid waiting time.

Despite the benefits using of the WAS, many people still registered via the usual queuing method. One reason we identified for not using the WAS was that over half of the participants did not know that an appointment can be obtained through the internet. This indicates that more effort should be made by hospitals and health service providers to promote and encourage the use of the WAS. By using a WAS, hospitals and other health service providers can reduce their invalid waiting time and anxiety in queues. Another important finding was that registration demand varied according to day of the week and time of the day. After investigating patient's appointment time in the WAS, we found that 43.8% of patients (2471) wanted to see a doctor on Monday or Tuesday, and 71.0% of them preferred it to be in the morning. This makes it very difficult to obtain registration during these periods. By making patients aware of this, it would be possible to encourage them to register on days with a lower outpatient load. This will help distribute the registration demand evenly and improve the allocation of medical resources.

Non-attendance is one of the potential problems for hospitals using a WAS. In our study, the overall non-attendance rate was 14.4%. This is consistent with the non-attendance rates reported in related studies, which range from 12% to 27% [10,11]. We found that the proportion of non-attendance was higher on Mondays than on other days, which differs from another study, which reported that non-attendance rates were higher on Sundays than on other days [12]. The failure of patients to meet scheduled appointments disrupts the orderliness of medical care and wastes limited medical resources [12,13]. Some measures had already been adopted to address this issue, such as preventing patients who failed to attend appointments three times in the preceding 12 months from making any new appointments on the web.

Many studies have focused on reasons for non-attendance, and some reliable suggestions for improvement have been provided (e.g., using a reminder system) [14-16]. To improve the management of outpatients, we assessed non-attendance related factors in our study. We found that hospital department, day of the week, and time of the day were significantly associated with non-attendance. The proportion of non-attendance was higher following registration with medical specialists (e.g. specialists in Neurosurgery) and most non-attendance occurred in the afternoon.

Our study has some potential limitations. First, we failed to collect data on the reasons for non-attendance from the participants using the WAS, because the majority of people who failed to attend their appointments declined to give an explanation. Second, because our study participants were from a large tertiary hospital, and different hospitals may have a different WAS, our findings may not reflect WAS use in other hospitals. Some relatively small hospitals may not need to use a WAS, because they do not experience high demand for registration. Despite these potential limitations, our study demonstrated that the WAS is an efficient and satisfactory means of obtaining registration, and identified factors that contribute to not using the booking system. By identifying these factors, we intend to develop intervention strategies to further improve the usability of the WAS and provide convenience for patients.

## Conclusion

Our study showed that use of a WAS can effectively increase patient satisfaction with getting a registration and reduce waiting times. A lack of information about online appointments was the main reason for not using the system. Non-attendance is an inevitable problem in the development of a web-based registration system. To increase the efficiency of the registration system, and to reduce non-attendance rates, further studies on various interventions such as the promotion of online registration, and use of a reminder system should be considered.

## Conflicts of interests

The authors declare that they have no competing interests.

## Authors' contributions

The initial idea was supplied by YW and YX. The protocol was drawn by YW and WC. Investigation was carried by DL, WC, HT, FS, ZT, CS and QY. Data extraction and analyses were performed by WC and YW. First draft was written by WC with contributions by YW. All authors commented and approved the final draft.

## Pre-publication history

The pre-publication history for this paper can be accessed here:

http://www.biomedcentral.com/1472-6963/11/318/prepub
